# Evaluating screening approaches for hepatocellular carcinoma in a cohort of HCV related cirrhosis patients from the Veteran’s Affairs Health Care System

**DOI:** 10.1186/s12874-017-0458-6

**Published:** 2018-01-04

**Authors:** Nabihah Tayob, Peter Richardson, Donna L. White, Xiaoying Yu, Jessica A. Davila, Fasiha Kanwal, Ziding Feng, Hashem B. El-Serag

**Affiliations:** 1 Department of Biostatistics, The University of Texas MD Anderson Cancer Center, Houston, USA; 2 Baylor College of Medicine, Houston, USA; 3 Michael E DeBakey VAMC HSR&D IQUEST, Houston, USA; 4 University of Texas Medical Branch at Galveston, Galveston, USA

**Keywords:** Early detection, Hepatocellular carcinoma, Longitudinal biomarkers, *α*-fetoprotein, Parametric empirical Bayes, Short-term risk prediction

## Abstract

**Background:**

Hepatocellular carcinoma (HCC) has limited treatment options in patients with advanced stage disease and early detection of HCC through surveillance programs is a key component towards reducing mortality. The current practice guidelines recommend that high-risk cirrhosis patients are screened every six months with ultrasonography but these are done in local hospitals with variable quality leading to disagreement about the benefit of HCC surveillance. The well-established diagnostic biomarker *α*-Fetoprotein (AFP) is used widely in screening but the reported performance varies widely across studies. We evaluate two biomarker screening approaches, a six-month risk prediction model and a parametric empirical Bayes (PEB) algorithm, in terms of their ability to improve the likelihood of early detection of HCC compared to current AFP alone when applied prospectively in a future study.

**Methods:**

We used electronic medical records from the Department of Veterans Affairs Hepatitis C Clinical Case Registry to construct our analysis cohort, which consists of serial AFP tests in 11,222 cirrhosis control patients and 902 HCC cases prior to their HCC diagnosis. The six-month risk prediction model incorporates routinely measured laboratory tests, age, the rate of change in AFP over the past year with the current AFP. The PEB algorithm incorporates prior AFP screening values to identify patients with a significant elevated level of AFP at their current screen. We split the analysis cohort into independent training and validation datasets. All model fitting and parameter estimation was performed using the training data and the algorithm performance was assessed by applying each approach to patients in the validation dataset.

**Results:**

When the screening-level false positive rate was set at 10%, the patient-level true positive rate using current AFP alone was 53.88% while the patient-level true positive rate for the six-month risk prediction model was 58.09% (4.21% increase) and PEB approach was 63.64% (9.76% increase). Both screening approaches identify a greater proportion of HCC cases earlier than using AFP alone.

**Conclusions:**

The two approaches show greater potential to improve early detection of HCC compared to using the current AFP only and are worthy of further study.

**Electronic supplementary material:**

The online version of this article (10.1186/s12874-017-0458-6) contains supplementary material, which is available to authorized users.

## Background

The incidence of hepatocellular carcinoma (HCC) in the United States has tripled over the last twenty years; however, the prognosis of patients diagnosed with HCC has remained poor with the five-year survival remaining less than 12% [1]. Patients with advanced stage HCC have few treatment options, with five-year survival between 0–10%, while those with early stage HCC have multiple treatment options (including surgical resection and liver transplantation), with 5-year survival for patients receiving these treatments > 60% [2]. Early detection of HCC through surveillance programs is a key component in reducing mortality.

The majority (80–90%) of HCC cases occur in patients with cirrhosis. Targeted cancer surveillance programs focus on those patients at high risk of disease and aim to increase the likelihood of early detection of cancer, while maintaining reasonable costs. The American Association for the Study of Liver Diseases (AASLD) recommends ultrasonography every six months in patients with cirrhosis [3]. The majority of surveillance ultrasounds in the United States take place in local hospitals with variable quality because ultrasonography is operator dependent, not sensitive in detecting early lesions and difficult to perform in obese patients. While ultrasonography has greater than 90% specificity, the reported sensitivity varies between 65–80%. Consequently there is disagreement in the field about the benefit of surveillance since there has been little evidence of improved survival in the few randomized clinical trials conducted. Considerable research has focused on developing highly sensitive standardized biomarker screening tests to complement (or replace) ultrasonography and provide motivation for HCC surveillance. A potential approach needs through vetting prior to being used in a prospective screening trial where the algorithm is used to trigger additional diagnosis work-ups.

Serum *α*-Fetoprotein (AFP) is a well established diagnostic biomarker for HCC that is widely used in screening despite the wide variation in its reported performance. A population-based US cohort study found that among HCC patients with a prior diagnosis of cirrhosis who received regular surveillance, 52% received both ultrasonography and AFP, 46% received AFP alone and 2% received ultrasonography alone [4]. A 2017 update of the AASLD guidelines recommends surveillance using ultrasonography, with or without AFP, every six months [5]. The sensitivity for AFP varies between 41–65% and the specificity between 80–95% in both diagnostic and screening settings and across a range of study designs when using a threshold of 20 ng/ml [6]. While other FDA approved biomarkers exist, it is unlikely that any other biomarker will be integrated into widespread HCC surveillance practice in the United States in the near future. Methods that improve the performance of the AFP, which can be used independently and in conjunction with ultrasonography, are critically needed in the short-term.

In this paper, we evaluate two approaches to improve the performance of AFP screening. The first incorporates routinely measured laboratory tests for evaluating the underlying liver disease of patients with cirrhosis and the rate of change in AFP in a six-month risk prediction model [7, 8]. The motivation behind this approach was driven by several studies that have explored the association between elevated AFP and other factors [9, 10]. In particular, Richardson et al. [11] found that in patients with no HCC, elevated AFP was associated with elevated alanine aminotransferase (ALT). Adjusting for these factors could improve the specificity of AFP in HCC surveillance. Since AFP is elevated in early stage HCC in only a subset of cases, including laboratory tests that monitor liver function could improve early detection of HCC.

The second approach is a parametric empirical Bayes (PEB) screening algorithm. The PEB method was first proposed by McIntosh & Urban [12] for cancer screening with a longitudinal biomarker. Previous algorithms for screening with longitudinal biomarkers, such as Skates et al. [13], required specifying the early pre-clinical behavior of the biomarker after disease onset in cases, which can be challenging when faced with limited serial data, as well as the biomarker trajectory in control patients. Patients whose biomarker trajectory to-date more closely resembles that of a case than a control patient were flagged as positive screens. In contrast, the PEB algorithm specifies the biomarker trajectory in control patients only, for whom there is often a great deal of data, and flags any significant deviations from the expected behavior given the model and the patient’s own serial history to date. The PEB algorithm has been applied to serial AFP data from the Hepatitis C Antiviral Long-term Treatment against Cirrhosis (HALT-C) trial [14]. In this randomized control trial, the PEB algorithm method improved the sensitivity of AFP by almost 17% compared to the standard thresholding approach (77.1% vs 60.4%) when the false positive rate among all screenings was set to 10%.

Our goal in this paper is to assess the performance of both the laboratory-based algorithm and the PEB algorithm for their ability to improve likelihood of early detection of HCC when applied prospectively in a future study. We consider (1) the sensitivity at fixed false positive rates during the entire screening period, within periods close to diagnosis, and within periods close to diagnosis while excluding intervals very close to clinical diagnosis where clinically significant earlier detection is unlikely; (2) the true positive rate, false positive rate, positive predictive value, and negative predictive value curves; and (3) the timing of first positive screen for each approach.

The most common etiology for cirrhosis in the United States is hepatitis C virus infection (HCV). The Department of Veterans Affairs (VA) is the largest integrated health-care provider in the United States and the veterans that utilize the VA are at high risk of HCV infection. The VA HCV Clinical Case Registry includes patient data for all HCV infected patients at 128 VA facilities and the detailed patient records and history contained in the HCV registry is possible as a result of the longstanding use of electronic medical records at VA facilitates. Current VA good practice guidelines recommend regular surveillance testing with ultrasonography and AFP at 6–12 month intervals in patients with cirrhosis. The VA HCV-cirrhosis cohort disease progression, variability of biomarkers under consideration and adherence to recommended screening visits more accurately reflects HCC screening in practice than a randomized control trial. In this analysis, we examine whether we can improve the likelihood of earlier detection of HCC in the regular clinical care setting, within the largest health care system in the United States, either by using routine blood tests in addition to current AFP levels in the laboratory-based algorithm or by using longitudinal AFP screening history via the PEB algorithm.

## Methods

### VA cohort construction

The VA HCV Clinical Case Registry includes patient demographic characteristics, laboratory test results, inpatient and outpatient visits, diagnostic and procedure codes, and date of death for all HCV-infected patients at VA facilities. All patients in our analysis cohort had a positive HCV antibody and HCV RNA test between 10/1/1997 and 9/30/2005. We used three ICD-9 diagnostic codes to identify patients with cirrhosis from the HCV cohort. The date of the first appearance of either 571.2, 571.5 or 571.6 in the electronic medical records was defined to be the cirrhosis diagnosis date. This definition has been validated and found to have 90% positive predictive value and 87% negative predictive value [15]. Our analysis cohort consisted of patients with a cirrhosis diagnosis at any time prior to end of study (12/31/2006). All the available patient information between the HCV index date (date of HCV diagnosis) and HCC diagnosis or end of study (12/31/2006) were included in the analysis dataset. In most patients (∼ 80%), the HCV index date preceded the cirrhosis diagnosis date and we chose to retain patient information between the HCV index date and the cirrhosis diagnosis date since the cirrhosis diagnosis is often delayed in these patients. If the HCV index date occurred after the cirrhosis diagnosis date, then it is likely that the cirrhosis diagnosis prompted the HCV testing.

The HCC diagnosis date was determined using both the ICD-9 codes and a subsequent manual structured review of the electronic medical records. First, we defined all patients with an ICD-9 code of 155.0 but without 155.1 to be probable HCC cases. A subset (∼ 82%) of these were manually reviewed and the date of HCC diagnosis was defined as the date of the earliest appearance of a liver mass on ultrasound that was subsequently confirmed by computed tomography (CT), magnetic resonance imaging (MRI), and/or biopsy, or in the absence of a mass on ultrasound, by the first evidence on CT, MRI and/or biopsy. We excluded patients who had ICD-9 codes that indicated an HCC diagnosis but had no confirmation of the HCC diagnosis in the manual review of the electronic medical records. The date of the first appearance of ICD-9 codes was defined to be the HCC diagnosis date in the small subset of HCC cases that were not manually reviewed.

**Fig. 1:**
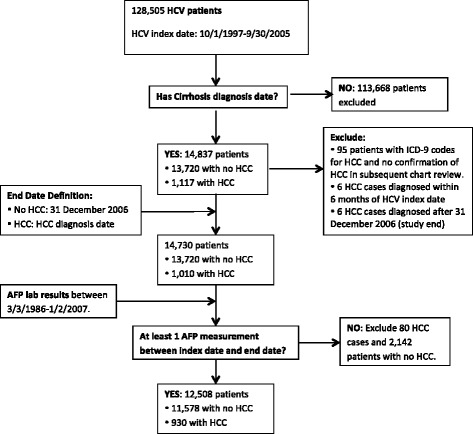
Standards for Reporting of Diagnostic accuracy (STARD) flow diagram for construction of the analysis cohort

Additional inclusion criteria used to create the analysis dataset were: at least one valid (> 0 ng/mL) serum AFP during the study period and HCC diagnosed at least 6 months after HCV index date and prior to the end of the study (12/31/2006). The analysis dataset at this stage consisted of 12,508 patients, of whom 930 have an HCC diagnosis during the study period. The dataset construction flow diagram is given in Fig. 1.

In order to obtain unbiased estimates of each algorithms performance, we split the analysis cohort into training and validation datasets. All model fitting and parameter estimation was performed using the training data and the algorithm performance was assessed by independently applying each approach to patients in the validation dataset. The training and validation cohorts were constructed by randomly dividing both the HCC cases and controls into two subgroups of equal sample size. Then one random sample of HCC cases and controls formed the training dataset and the other random sample of HCC cases and controls formed the validation dataset.

We used the following notation in the description of the both screening algorithms. Suppose that there are *N*_*T*_ patients in the training dataset used to fit the model and *N*_*V*_ patients in the validation dataset used to assess the model performance. Without loss of generality, we assume all time is measured from the HCV index date. In the notation that follows, the subscript *i*=1,…,*N*_*T*_ indexes the training dataset patients and *i*=*N*_*T*_+1,…,*N*_*T*_+*N*_*V*_ indexes the validation dataset patients. For those patients diagnosed with HCC during the study (i.e., cases), let *δ*_*i*_=1 and *d*_*i*_ be the months to HCC diagnosis since HCV index date. For patients not diagnosed with HCC during the study (i.e., controls), let *δ*_*i*_=0 and *d*_*i*_ be the months to the end of study since HCV index date.

### Screening algorithms

The standard approach to screening with AFP is to compare the biomarker level at each screening time to a fixed threshold. In our paper, we explore two approaches to screening that incorporate information beyond the current AFP level at each screening visit.

#### Laboratory-based algorithm

The first is based on using a short term risk prediction model that incorporates AFP, the rate of change in AFP in the past year (if available), other laboratory tests and demographic variables to determine which patients are at high risk of developing HCC in the short term. The risk score from this model can then be used to determine which patients should be sent for further imaging because they could possibly have HCC.

El-Serag et al. [7] considered laboratory tests that are widely used, standardized, reproducible and clearly defined in the electronic medical records for inclusion in their six-month risk prediction model. The final model included AFP, ALT, platelets (PLT), age and two-way interactions between AFP and ALT and AFP and PLT. Logarithmic transformations and spline functions were used to model the nonlinear relationship between the included covariates and six-month probability of HCC. This model selection was done using the same cohort of patients that we are using in this study. Therefore, while we have attempted to reduce the bias from overfitting by splitting the VA cohort into training and testing dataset, we will require a new cohort of patients to get truly independent validation of the performance results for this proposed laboratory-based algorithm. In White et al. [8], the six-month risk prediction model was updated to include the rate of change in AFP in the past year, since it has been shown that both the current AFP levels and the trajectory of AFP are predictive of HCC [16]. Since not all patients will have an AFP measurement in the prior year, we have adapted their model to include change in AFP in the last year when it is available.

We use laboratory tests extracted from the electronic medical record to estimate the model parameters and assess the performance of the proposed laboratory-based algorithm. We consider each AFP test date to be a screening visit. It is unlikely that all the patients will have the other tests (ALT, PLT) performed on the same day; in practice these tests will not be re-run if they were recently performed. We considered any ALT or PLT laboratory test within 6 months prior to the AFP test to be a valid concurrent lab test.

A cross-sectional re-sampling approach was used to estimate the predicted probability of HCC. For each patient in the training dataset, a random screening visit was chosen from all possible screenings and the six-month risk prediction model was fit using a logistic regression model. This process was repeated 100 times and the parameter estimates from each iteration were saved. For each new patient, the predicted probability of HCC within six months is calculated by averaging the 100 estimates of the predicted probability of HCC within six-months based on the parameter estimates from each iteration. The full details of the approach are described below.

Each patient has *n*_*i*_ AFP tests performed at screening visits {*t*_*ij*_,*j*=1,…,*n*_*i*_}. We define an indicator variable for each screening visit that is 1 if the patient is diagnosed with HCC within six months of that visit and 0 otherwise. i.e. At the *j*^*th*^ screening visit for the *i*^*th*^ patient, *D*_*ij*_=1 if *d*_*i*_<(*t*_*ij*_+6) and *δ*_*i*_=1 and 0 otherwise. For the *i*^*th*^ patient at the *j*^*th*^ screening visit, we extract *AFP*_*ij*_=AFP level in ng/ml, *ALT*_*ij*_=most recent ALT level in IU/ml measured within the interval [*t*_*ij*_−6,*t*_*ij*_], *PLT*_*ij*_= most recent PLT level in 1000’s measured within the interval [*t*_*ij*_−6,*t*_*ij*_] and *Age*_*ij*_= age in years at AFP test. Note that *AFP*_*ij*_, *ALT*_*ij*_ and *PLT*_*ij*_ are truncated at 1. We define an indicator function $\Delta AFP_{ij}^{obs}$ that is 1 if *t*_*ij*_−*t*_*i*(*j*−1)_≤12, i.e. the previous AFP measurement was within the last year, and 0 otherwise. The AFP rate of change in the previous year is defined to be log2(*ΔAFP*_*ij*_)=[ log2(*AFP*_*ij*_)− log2{*AFP*_*i*(*j*−1)_}]/[{*t*_*ij*_−*t*_*i*(*j*−1)_}/12].

The six-month risk prediction model is

1 $$\begin{array}{@{}rcl@{}}  \log\! \left\{\! \frac{Pr\left(D_{ij}=1 | AFP_{ij}, AFP_{i(j-1)}, Age_{ij}, ALT_{ij}, PLT_{ij}\right)}{Pr\left(D_{ij}=0 | AFP_{ij}, AFP_{i(j-1)}, Age_{ij}, ALT_{ij}, PLT_{ij}\right)} \!\right\} \!\!= \!\beta_{1} \mathbf{X}_{ij}^{T} \end{array} $$

where *β*_1_ is the row vector containing the model parameters and **X**_*ij*_ is defined to be the row vector $[\mathbf {AFP}_{ij},\mathbf {ALT}_{ij},\mathbf {PLT}_{ij},\mathbf {Age}_{ij},\mathbf {AFP}_{ij}*\mathbf {ALT}_{ij},\noindent \mathbf {AFP}_{ij}*\mathbf {PLT}_{ij}, 1-\Delta AFP_{ij}^{obs}, \mathbf {\Delta AFP}_{ij}]$ with

$${{}\begin{aligned} \mathbf{AFP}_{ij} &=\left[\log_{2}\left(AFP_{ij}\right), \left\{\log_{2}\left(AFP_{ij}\right)-2\right\}*I\left\{\log_{2}\left(AFP_{ij}\right)>2\right\},\right.\\ &\quad \left.\left\{\log_{2}\left(AFP_{ij}\right)-7\right\}*I\left\{\log_{2}\left(AFP_{ij}\right)>7\right\}\right.,\\ &\quad \left.\left\{\log_{2}\left(AFP_{ij}\right)-9\right\}*I\left\{\log_{2}\left(AFP_{ij}\right)>9\right\}\right]\\ \mathbf{ALT}_{ij}&=\left[\log_{2}\left(ALT_{ij}\right), \left\{\log_{2}\left(ALT_{ij}\right)-\log_{2}(20)\right\}*I\left\{\log_{2}\left(ALT_{ij}\right)\right.\right. \\ &\qquad \left.\left. >\log_{2}(20)\right\} \right.\\ &\quad\left.\left\{\log_{2}\left(ALT_{ij}\right)-\log_{2}(50)\right\}*I\left\{\log_{2}\left(ALT_{ij}\right)>\log_{2}(50)\right\},\right.\\ &\quad\ \left\{\log_{2}\left(ALT_{ij}\right)-\log_{2}(100)\right\}*\\ &\quad\left.I\left\{\log_{2}\left(ALT_{ij}\right)>\log_{2}(100)\right\}, \left\{\log_{2}\left(ALT_{ij}\right)\! -\! \log_{2}(200)\right\}\right. \\ & \qquad \left. *I\left\{\log_{2}\left(ALT_{ij}\right)>\log_{2}(200)\right\}\right] \\ \mathbf{PLT}_{ij}&=\left[PLT_{ij}, \left(PLT_{ij}-35\right)*I\left(PLT_{ij}>35\right)\right] \\ \mathbf{Age}_{ij}&=\left[Age_{ij}, \left(Age_{ij}-50\right)*I\left(Age_{ij}>50\right)\right]\\ \mathbf{\Delta AFP}_{ij}&=\Delta AFP_{ij}^{obs}*\left[\log_{2}\left(\Delta AFP_{ij}\right), \left\{\log_{2}\left(\Delta AFP_{ij}\right)+8\right\}\right. \\ &\qquad \left. *I\left\{\log_{2}\left(\Delta AFP_{ij}\right)>-8\right\}, \right.\\ &\quad \left\{\log_{2}\left(\Delta AFP_{ij}\right)-0\right\}*I\left\{\log_{2}\left(\Delta AFP_{ij}\right)>0\right\}, \\ &\quad \left\{\log_{2}\left(\Delta AFP_{ij}\right)-2\right\}*I\left\{\log_{2}\left(\Delta AFP_{ij}\right)>2\right\},\\ &\quad \left.\left\{\log_{2}\left(\Delta AFP_{ij}\right)-9\right\}*I\left\{\log_{2}\left(\Delta AFP_{ij}\right)>9\right\}\right]. \end{aligned}} $$

Note that *I*(·) is an indicator function that takes the value 1 when the argument is true and 0 when the argument is false.

The cross-sectional re-sampling algorithm used to estimate the predicted probability of HCC for a each patient in the validation cohort is:

- For each *k*=1,…,100,
  1. Create *k*^*th*^ cross-sectional draw from longitudinal training data: for each patient draw a random visit *t*_*ij*_ from {*t*_*ij*_,*j*=1,…,*n*_*i*_} with replacement, *i*=1,…,*N*_*T*_.
  2. Fit logistic regression model (1) to get parameter estimates $\hat {\beta _{1}}_{k}$.
- The predicted probability of HCC within six-months at the *j*^*th*^ screening visit for the *i*^*th*^ patient (*i*=*N*_*T*_+1,…,*N*_*T*_+*N*_*V*_) is
  $$\begin{array}{@{}rcl@{}} \eta_{ij}=\frac{1}{100} \sum_{k=1}^{100} \frac{\exp\left(\hat{\beta_1}_{k} \mathbf{X}_{ij}^{T}\right)}{1+\exp\left(\hat{\beta_1}_{k} \mathbf{X}_{ij}^{T}\right)} \end{array} $$

The laboratory-based algorithm will indicate a positive screen if *η*_*ij*_ exceeds a pre-specified threshold *c*.

#### Parametric empirical Bayes algorithm

The second approach was proposed by McIntosh and Urban [12] and incorporates the longitudinal history of screening biomarker to define subject and screen specific thresholds. The defining feature of a useful screening biomarker is that it is predictable or stable in the absence of disease and exhibits a characteristic change after disease onset. For these biomarkers, the PEB algorithm incorporates the known information about the variability of longitudinal biomarker measurements within a patient and between patients to detect smaller but significant increases in the biomarker. In addition, the PEB algorithm may reduce the number of false positive screens in patients with no disease and a stable biomarker trajectory that is higher than average since it has the ability to learn from prior false positive screens.

Let *Y*_*ij*_= log2(*AFP*_*ij*_) be the transformed AFP level in the *i*^*th*^ patient at the *j*^*th*^ screen. The PEB approach assumes the following hierarchical model to describe the distribution of the transformed biomarker in the population of control patients.

$$\begin{array}{@{}rcl@{}} Y_{ij}|\theta_{i} \sim N\left(\theta_{i},\sigma^{2}\right) \\ \theta_{i} \sim N\left(\bar{\theta},\tau^{2}\right) \end{array} $$

I.e. given the patient-specific mean *θ*_*i*_, the transformed biomarker levels *Y*_*ij*_ are independent and identically distributed with mean *θ*_*i*_ and variance *σ*^2^ and *θ*_*i*_ itself is normally distributed with mean $\bar {\theta }$ and variance *τ*^2^. The within-subject variance *σ*^2^ and between-subject variance *τ*^2^ are key measures that affect the performance of the PEB algorithm. *Y*_*ij*_ can be centered and rescaled to simplify the derivation. Let $Z_{ij}=(Y_{ij}-\bar {\theta })/\sqrt {\sigma ^{2} + \tau ^{2}}$. Then

$$\begin{array}{@{}rcl@{}} Z_{ij}|\mu_{i} \sim N\left(\mu_{i},1-B_{1}\right) \\ \mu_{i} \sim N(0,B_{1}) \\ \text{where}\ B_{1}=\frac{\tau^{2}}{\sigma^{2} + \tau^{2}}. \end{array} $$

Note that a simple calculation verifies that the marginal distribution of *Z*_*ij*_ is the standard normal distribution. The PEB algorithm can be modified using different distributional assumptions but we continue with the original formulation of the approach for two reasons. Firstly, screening rules are invariant to monotonic transformation so for any continuous marker, a transformation to normality is assured and secondly, the hierarchical normal model results in simplified derivations and calculations. In the implementation of the PEB algorithm, standard tests of normality can be used to evaluate whether the chosen transformation is appropriate.

The standard threshold approach ignores prior screening history of the patient and instead uses the same threshold for all patients. One possible approach for determining this threshold is to use the above model, which describes the transformed biomarker distribution in the control population, to specify a threshold that controls the population-wide false positive rate (FPR). Since *Z*_*ij*_ is assumed to follow a standard normal distribution, then $\phantom {\dot {i}\!}Pr(Z_{ij} > z_{1-f_{0}})=f_{0}$ where $z_{1-f_{0}}$ is the 100(1−*f*_0_) percentile of the standard normal distribution. Therefore, using the standard threshold screening rule, patient *i* has a positive screen at the *j*^*th*^ screening visit if $\phantom {\dot {i}\!}Z_{ij} > z_{1-f_{0}}$.

If the patient’s mean biomarker level (*μ*_*i*_) were known, we could define an individually tailored screening rule that still ensures the population-wide FPR is not more than *f*_0_ since given *μ*_*i*_, $(Z_{ij}-\mu _{i})/\sqrt {1-B_{1}}$ follows a standard normal distribution. Therefore $Pr\{(Z_{ij}-\mu _{i})/\sqrt {1-B_{1}} > z_{1-f_{0}} | \mu _{i}\}=f_{0}$ and patient *i* has a positive screen at the *j*^*th*^ screening visit if $Z_{ij} > \mu _{i} + z_{1-f_{0}}\sqrt {1-B_{1}}$.

However *μ*_*i*_ is not known, so instead we use the PEB estimate of this parameter. This estimate, denoted by $\hat {\mu }_{ij}$, is a weighted average of the population mean (which is 0 in this case) and the sample average of the patients screening history. The PEB screening rule then indicates a positive screen for patient *i* at the *j*^*th*^ screening visit if

2 $$\begin{array}{@{}rcl@{}} Z_{ij} > \hat{\mu}_{ij} + z_{1-f_{0}}\sqrt{1-B_{1} B_{j}}, \end{array} $$

where $\hat {\mu }_{ij} = 0*(1-B_{j}) + \bar {Z}_{ij}*B_{j}$, $\bar {Z}_{ij}=\frac {1}{j-1}\sum _{j'=1}^{j-1} Z_{ij'}$ and $B_{j}=\frac {\tau ^{2}}{\sigma ^{2}/(j-1) + \tau ^{2}}$.

To implement the PEB screening algorithm, we require estimates for the parameters $\bar {\theta }$, *σ*^2^ and *τ*^2^. These can be obtained by fitting a linear mixed model with a random intercept in the control patients from the training cohort. We then apply the PEB screening rule to all the screenings conducted in the validation cohort.

#### Incorporation of an OR rule

In clinical practice, if the current AFP level is very high (e.g. *AFP*_*ij*_≥400 ng/ml) then the patient will automatically be sent for follow-up imaging with CT or MRI and no additional screening algorithm will be used. To formalize this practice, we include an OR rule in the implementation of both the screening algorithms in the validation dataset. This approach is called an OR rule since patient *i* has a positive screen at time *t*_*ij*_ if *AFP*_*ij*_≥400 ng/ml or if the screening algorithm indicates a positive screen. We define a general variable *P*_*ij*_(·) that is 1 when patient *i* has a positive screen at time *t*_*ij*_ and 0 otherwise and is a function of the thresholding parameter for each algorithm. For the laboratory-based algorithm, *P*_*ij*_(·) is defined to be

$$\begin{array}{@{}rcl@{}} P_{ij}(c)=I\left(AFP_{ij}\geq400~\text{or~} \eta_{ij}>c\right) \end{array} $$

and for the PEB algorithm it is

$$\begin{array}{@{}rcl@{}} P_{ij}\left(1-f_{0}\right)=I\left\{AFP_{ij}\geq400~\text{or~} \Phi \left(\frac{Z_{ij}-\hat{\mu}_{ij}}{\sqrt{1-B_{1} B_{j}}} \right)> 1-f_{0}\right\}, \end{array} $$

where *Φ* is the standard normal cumulative distribution function. The threshold of 400 ng/ml for AFP was chosen because it corresponds to a very low false positive rate (0.006) in our training dataset.

### Evaluation of screening algorithms

The standard measures used to evaluate the performance of biomarker screening approaches are based on screening at a single time point. For example, sensitivity is proportion of cases with a positive test and specificity is proportion of controls with a negative test. We have extended these definitions to the longitudinal screening setting. In patients not diagnosed with HCC during the study period, it is clear that any negative screening result is a true negative, while any positive screening result is a false positive screen. However in patients diagnosed with HCC, we do not know when the cancer started developing, we only know when it was clinically diagnosed. Therefore, we consider multiple possible definitions for sensitivity and specificity in the longitudinal setting that are all dependent on which screenings in HCC cases are considered true positive screens and which are considered false positive screens because its unlikely that additional imaging with CT or MRI would have resulted in detection of HCC at that time.

**Fig. 2:**
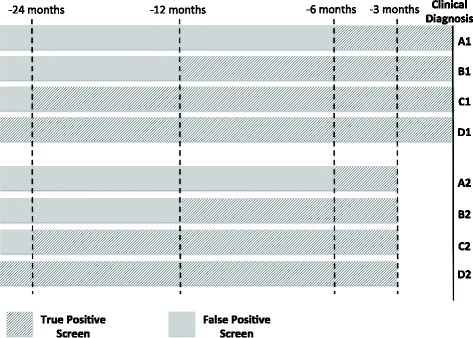
Visual illustration of different possible periods during which a positive screening result was considered to be a true positive screen and periods where a positive screen result was considered to be a false positive screen in HCC cases

In Fig. 2 we illustrate these definitions where we progressively increase the time period prior to clinical diagnosis during which positive screens in HCC cases were considered to be true positive screens. In definitions A1-D1, we considered all screenings prior to clinical diagnosis of HCC when calculating the patient-level sensitivity. In definitions A2-D2, we excluded screenings within three months of clinical diagnosis of HCC when calculating the patient-level sensitivity since the goal of the screening algorithms are to increase the earlier detection of HCC and a positive screen result within three months of the clinical diagnosis of HCC is unlikely to result in a clinically significant difference in the prognosis for a patient.

We then define patient-level sensitivity or true positive rate (TPR) as the probability of an HCC case having at least one positive screen during the specified pre-clinical detection period indicated in Fig. 2:

$${}\begin{aligned} TPR\left(\cdot,\tau_{1},\tau_{2}\right)&=\text{Pr} \left\{ \text{at least one}\ P_{ij}(\cdot)=1, j=1,\ldots,n_{i} | d_{i}-\tau_{1} \leq t_{ij}\right.\\ &\left.\quad \leq d_{i}-\tau_{2}, \delta_{i}=1 \right\}, \end{aligned} $$

where *τ*_1_ and *τ*_2_ define the boundaries within which a positive screen was considered to be a true positive. For example, in definition A1, *τ*_1_=6 months and *τ*_2_=0 months and in definition A2, *τ*_1_=6 months and *τ*_2_=3. We defined sensitivity at the patient-level because the goal is to assess the future performance of the algorithm in terms of the number of HCC cases that could be detected prior to clinical diagnosis. In the future, a single positive screen that leads to confirmation of HCC via additional imaging would terminate further screening.

Screening-level FPR (1-specificity), was defined as the probability of a positive screen among (1) all the screenings conducted in the control patients and (2) the screenings conducted in HCC cases that are considered to be outside the detection period indicated in Fig. 2:

$$\begin{array}{@{}rcl@{}} FPR\left(\cdot,\tau_{1}\right)=\text{Pr} \left\{P_{ij}(\cdot)=1 | \delta_{i}=1 \text{~and~} t_{ij}<d_{i}-\tau_{1} \text{~or~} \delta_{i}=0 \right\}. \end{array} $$

The FPR was defined at the screening level because each false positive result would lead to further testing that can be expensive and may increase the likelihood of complications and anxiety.

The positive predictive value (PPV) was defined as the probability of positive screen occurring in an HCC case within the specified pre-clinical detection period indicated in Fig. 2:

$$\begin{array}{@{}rcl@{}} PPV\left(\cdot,\tau_{1},\tau_{2}\right)=\text{Pr} \left\{d_{i}-\tau_{1} \leq t_{ij} \leq d_{i}-\tau_{2}, \delta_{i}=1 | P_{ij}(\cdot)=1 \right\}. \end{array} $$

This measure was reported at the screening-level because the goal is to evaluate the probability of any positive screen being a true positive.

The negative predictive value (NPV) was defined as the probability of negative screen occurring in (1) control patients or (2) in HCC cases that are considered to be outside the detection period indicated in Fig. 2:

$$\begin{array}{@{}rcl@{}} NPV(\cdot,\tau_{1})=\text{Pr} \left\{\delta_{i}=1~\text{and~} t_{ij}<d_{i}-\tau_{1}~\text{or~} \delta_{i}=0 | P_{ij}(\cdot)=0 \right\}. \end{array} $$

This measure was reported at the screening-level because the goal is to evaluate the probability of any negative screen being a true negative. Note that both the PPV and NPV measures are influenced by the prevalence of HCC in our analysis cohort as well as the number of screenings conducted in patients. In Additional file 1: Appendix A, we provide estimators of the four measures that we used in our analysis.

## Results

Of the 12,508 patients in the analysis cohort, 12,124 had at least one AFP test with both an ALT and PLT laboratory test performed within the prior six months. This cohort of patients was randomly split into the training and validation cohorts each consisting of 451 HCC cases and 5611 controls. Our goal is to assess the performance of each of the screening algorithms within the OR rule, i.e. the patient has a positive screen if either *AFP*≥400 ng/ml or the screening algorithm indicates a positive screen. Therefore the training cohort was further restricted to only those with *AFP*<400 ng/ml since the screening algorithms will only be applied in those patients. We do not restrict the validation cohort since our goal is to assess the performance of the screening algorithms as they would be used in clinical practice, which includes the OR rule. Note that in our analysis we have patients with multiple laboratory tests on the same day. For these patients, the multiple laboratory tests on the same day were summarized (average of the log2 measurements) and this value was used in the analysis.

In Table 1 we describe the training and validation cohorts. Across the cohorts, we observe that age at baseline (first AFP test), the proportion of white and black patients, the months between AFP tests and the baseline AFP, ALT and PLT were all similar within controls and HCC cases. In control patients, baseline AFP and ALT were slightly lower and baseline PLT was slightly higher compared to those patients eventually diagnosed with HCC. The average screening interval was around 12 months. Approximately 28% of the patients had only a single AFP test during the study, while ∼ 22% had more than four AFP tests during the study.

**Table 1:** Demographic table for training and validation HCV-related cirrhosis cohorts

	Training cohort		Validation cohort	
	Controls	HCC cases	Controls	HCC cases
N	5611	451	5611	451
Age at baseline (years)	52.87 (7.28)	54.67 (7.78)	52.93 (7.39)	54.89 (7.49)
Male	5498 (97.99%)	449 (99.56%)	5501 (98.04%)	447 (99.11%)
Female	113 (2.01%)	2 (0.44%)	110 (1.96%)	4 (0.89%)
Race				
White	1954 (34.82%)	173 (38.36%)	2034 (36.25%)	171 (37.92%)
Black	623 (11.10%)	54 (11.97%)	576 (10.27%)	60 (13.30%)
Other/Unknown	3034 (54.07%)	224 (49.67%)	3001 (53.48%)	220 (48.78%)
Number of AFP tests				
1	1598 (28.48%)	116 (25.72%)	1598 (28.48%)	138 (30.60%)
2	1297 (23.12%)	119 (26.39%)	1240 (22.10%)	106 (23.50%)
3-4	1480 (26.38%)	119 (26.39%)	1524 (27.16%)	120 (26.61%)
> 4	1236 (22.03%)	97 (21.51%)	1249 (22.26%)	87 (19.29%)
Months between AFP tests	11.67 (11.00)	10.30 (10.10)	11.83 (11.26)	10.93 (10.89)
Baseline AFP in log2(ng/ml)	2.92 (1.55)	4.72 (2.78)	2.95 (1.57)	4.57 (2.95)
Baseline ALT in log2(ng/ml)	6.14 (1.03)	6.31 (0.91)	6.16 (1.05)	6.35 (0.88)
Baseline PLT in 1000’s	147.97 (78.88)	123.50 (66.06)	148.51 (78.83)	127.97 (76.08)

For continuous variables we report means and standard deviations in parenthesis and for categorical variables we report the number in each group and percentages in parenthesis. Baseline was defined to be the date of the first AFP test

In the tables and figures, the “AFP only” approach is a six-month risk prediction model with AFP only, the laboratory-based algorithm is referred to as the “AFP+Lab+ *Δ*AFP” algorithm and the PEB algorithm applied to AFP is referred to as the “PEB: AFP” approach. In the first comparison of the screening algorithms, we focused on the patient-level TPR when the screening-level FPR was fixed. In Table 2, the screening-level FPR was fixed at 10% and in Table A in Additional file 1: Appendix B, the screening-level FPR was fixed at 5%. We observe that both the laboratory-based algorithm and the PEB approach show improved TPR over the standard thresholding approach with AFP only across all the definitions of true positive screenings in HCC cases (A1-D1 and A2-D2). The TPR of the PEB algorithm was 9.75% greater than the standard thresholding approach with AFP only (63.64% vs 53.88%) and 5.55% greater than the AFP+Lab+ *Δ*AFP approach (63.64% vs 58.09%) over the entire screening period (definition D1) and 5.81% greater than the standard thresholding approach with AFP only (60.23% vs 54.42%) and 1.16% greater than the AFP+Lab+ *Δ*AFP approach (60.23% vs 59.07%) in the two-years prior to clinical diagnosis (defintion C1).

**Table 2:** Comparison of the patient-level true positive rate (*TPR*(·,*τ*_1_,*τ*_2_)) when the threshold for each screening algorithm was chosen such that the screening-level false positive rate is 10%, i.e *FPR*(·,*τ*_1_)=0.1. In each definition, the choice of the parameters *τ*_1_ and *τ*_2_ varies

	Results from validation cohort							
Screening algorithm	A1	B1	C1	D1	A2	B2	C2	D2
AFP only	0.5753	0.5672	0.5442	0.5388	0.4019	0.4099	0.3564	0.3361
AFP+Lab+ *Δ*AFP	0.6137	0.6119	0.5907	0.5809	0.4766	0.4820	0.4158	0.3770
PEB: AFP	0.6055	0.6045	0.6023	0.6364	0.4579	0.4955	0.4653	0.4891
Number of HCC cases	365	402	430	451	107	222	303	366

A1: *τ*_1_=6 months and *τ*_2_=0, B1: *τ*_1_=12 months and *τ*_2_=0, C1: *τ*_1_=24 months and *τ*_2_=0, D1: *τ*_1_ is the maximum follow-up time and *τ*_2_=0. A2: *τ*_1_=6 months and *τ*_2_=3 months, B2: *τ*_1_=12 months and *τ*_2_=3 months, C2: *τ*_1_=24 months and *τ*_2_=3 months, D2: *τ*_1_ is the maximum follow-up time and *τ*_2_=3 months. AFP+Lab+ *Δ*AFP: updated laboratory-based algorithm, PEB: AFP: parametric empirical Bayes algorithm applied to AFP

When the screening-level FPR is fixed at 5% (Table A in Additional file 1: Appendix B), we observe that the PEB algorithm outperforms the other approaches implemented for all the definitions of true positive screenings in HCC cases except when comparing the PEB approach to the AFP+Lab+ *Δ*AFP approach in the three to six and three to twelve months prior to clinical diagnosis (defintion A2 and B2). In the remaining analyses we focus on 10% screening-level FPR because HCC screening is performed in high-risk cirrhosis patients and therefore we can allow for a higher number of false positive screenings. In our validation cohort a 10% screening-level FPR corresponds to a fixed threshold of 35.7 ng/ml for AFP in the standard approach based on definition D1. This was higher than the most commonly used threshold for AFP of 20 ng/ml, which would have a higher screening-level FPR.

We chose a split-sample approach with training and validation cohorts to evaluate our HCC screening algorithms since we have a large cohort with 902 HCC cases and 11,222 controls. In a sensitivity analysis, we utilized an out-of-bag bootstrap validation approach, where each bootstrap training cohort consisted of 12,124 patients drawn with replacement from the full analysis cohort and each bootstrap validation cohort consisted of all the patients not included in the bootstrap training cohort. The model parameters for each of the HCC screening algorithms were estimated using the training cohort, the screening algorithms were implemented in the validation cohort and the patient-level TPR at 10% screening-level FPR was estimated. This procedure was repeated 300 times and the results were averaged over the bootstrap iterations. In Table B in Additional file 1: Appendix B, we observe that the results are mostly consistent; both the laboratory-based algorithm and the PEB approach showing improved TPR over the standard thresholding approach with AFP only across all the definitions of true positive screenings in HCC cases except one. For definition A2 with a restrictive time frame (only positive screens within 3–6 months prior to HCC diagnosis are true positives) and fewer HCC cases, the PEB algorithm and AFP only algorithm are approximately equivalent. In the two-years prior to HCC diagnosis (definition C1), the TPR of the PEB algorithm was 5.03% greater than the standard thresholding approach with AFP only (61.26% vs 56.23%) and 1.57% greater than the AFP+Lab+ *Δ*AFP approach (61.26% vs 59.69%).

**Fig. 3:**
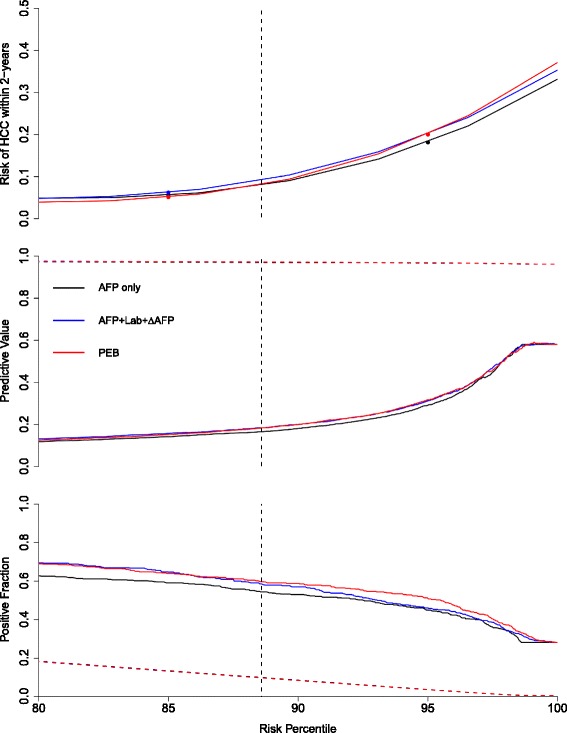
Comparison of screening algorithms within two years of clinical diagnosis (C1 in Fig. 2). In the top panel, we plot the estimated risk of HCC within two years for each screening approach against corresponding the risk percentile, which is defined to be the corresponding proportion of screens that lie below the threshold. The middle panel displays the positive predictive value (*PPV*(·,*τ*_1_=24,*τ*_2_=0), solid line) and the negative predictive value (*NPV*(·,*τ*_1_=24), dashed line) against the risk percentile and the bottom panel displays the patient-level true positive fraction (*TPR*(·,*τ*_1_=24,*τ*_2_=0), solid line) and the screening-level false positive fraction (*FPR*(·,*τ*_1_=24), dashed line) against the risk percentile. The vertical dashed lines in each plot correspond to the risk percentile associated with 10% screening-level FPR. The figures focus on curves between the 80th and 100th risk percentile. AFP+Lab+ *Δ*AFP: updated laboratory-based algorithm, PEB: parametric empirical Bayes algorithm applied to AFP

In Fig. 3, we compared the patient-level TPR, screening-level FPR, PPV and NPV curves of the screening algorithms when only positive screens within two years of clinical diagnosis were considered to be true positive screens (definition C1). We defined the four measures in “Evaluation of screening algorithms” section, where all measures are functions of the thresholding parameter for each screening algorithm (*c* or 1−*f*_0_). In order to standardize the curves for each approach, we redefined each measure to be a function of the risk percentile: the proportion of screens that lie below *c* or 1−*f*_0_. In addition, we estimated the risk of HCC within *τ*_1_=24 months for each decile. I.e. we estimated the probability of being diagnosed with HCC within the next two years, given that a patient’s current screen places them within the *k*^*th*^ decile, for *k*=1,…,10. In Fig. 3, we used a cubic spline to create the estimated risk curve.

We observed small differences in the PPV and NPV curves across the screening algorithms (middle panel of Fig. 3). In the bottom panel of Fig. 3, we observed the screening-level FPR had a linear relationship with the risk percentile that was the same for each approach (by definition) and that there was separation between the patient-level TPR across the different methods.

The structure of Fig. 3 allows for comparison across the different methods and conveys a great deal of information. For example, we illustrate how to extract the results for Table 2 from these curves in Fig. 3. In the bottom panel, we fix the screening-level FPR at 0.1 and find the corresponding risk percentile. Using vertical dashed lines in each panel, we can extract the patient-level TPR and PPV and NPV as well as the corresponding estimate of the risk of HCC within two years for each algorithm. Alternatively, we could fix any other measure at a pre-specified level and compare the screening algorithms with respect to the remaining measures. In Additional file 1: Appendix B, we include the corresponding figures for definitions A1, B1 and D1 in Figures A, B and C respectively.

Next, we evaluated the screening algorithms at the individual patient level. When we considered all positive screens more than two years prior to clinical diagnosis in HCC cases to be false positive screens (definition C1) and fixed the screening-level FPR at 10%, we observed that 4.15%, 3.93% and 1.79% of patients have more than two false positive screening using the AFP only, AFP+Lab+ *Δ*AFP and PEB approach respectively. Since we have fixed the number of false positive screenings allowed in each method, this illustrates how each method distributes the number of false positive screens across the patients and reveals one of the advantages of the PEB approach— the ability to learn from prior false positive screens and reduce the number of false positive screens in an individual patient.

**Fig. 4:**
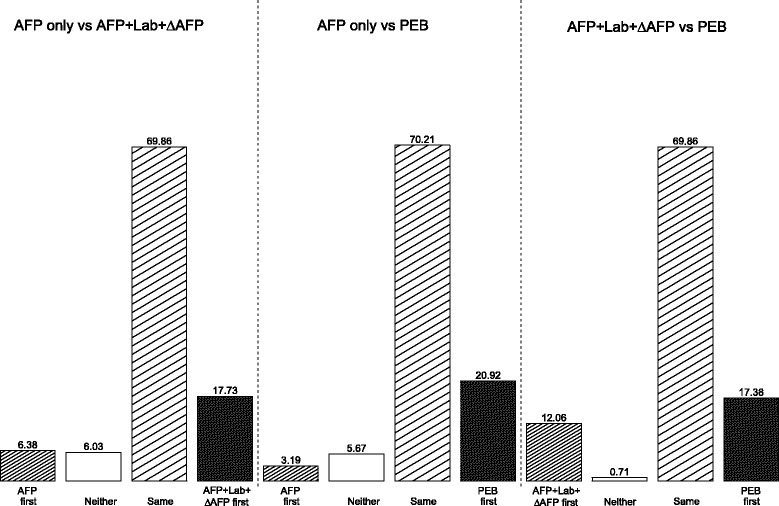
Comparison of first positive screen time between methods in the validation cohort within 2 years of clinical diagnosis (C1 in Fig. 2). AFP+Lab+ *Δ*AFP: updated laboratory-based algorithm, PEB: parametric empirical Bayes algorithm applied to AFP

Among the 430 HCC cases with screenings in the two years prior to clinical diagnosis, 282 had at least one positive screen and 148 had no positive screening for any of the approaches during this period. In Fig. 4, we compare the timing of the first positive screens in the 282 HCC cases that were flagged positive by at least one screening algorithm. The time of the first positive screen for any screening algorithm with no positive screens during the two-years prior to clinical diagnosis was defined to be the clinical diagnosis time. In the first panel, we compared the AFP only approach to the AFP+Lab+ *Δ*AFP algorithm and observed that while 69.86% of the HCC cases were first flagged positive at the same screening visit, 17.73% were flagged first by the AFP+Lab+ *Δ*AFP algorithm compared to the 6.38% that were first flagged positive by the AFP only approach. In the middle panel, we observe that while a similar proportion of the HCC cases were first flagged positive at the same time by both the AFP only approach and the PEB approach (70.21%), 20.92% were flagged first by the PEB algorithm while only 3.19% were first flagged positive by the AFP only approach. The earlier positive screens for the PEB approach were demonstrated in the third panel, which compared the PEB approach to the AFP+Lab+ *Δ*AFP algorithm.

## Discussion

We have evaluated multiple approaches for HCC screening in a cohort of active HCV-related cirrhosis patients from the VA patient population between 1997 and 2006. Each of the approaches under consideration included information beyond the current AFP level to increase the number of patients that are flagged with positive screens. Across all the analyses, we observed that including additional widely available and objective information leads to improvements in HCC screening performance measures. The goal of HCC screening is to detect HCC earlier, when there are potentially more curative treatment options available to the patient, and screening algorithms that have positive screens in the one to two years prior to clinical diagnosis of HCC are more likely to lead to earlier detection of HCC.

The performance of the PEB algorithm is affected by both the variability of AFP within and between patients. In the HALT-C Trial, a clinical trial with strict patient inclusion criteria, the PEB algorithm improved the sensitivity of AFP by 16.7% compared to the standard thresholding approach (77.1% vs 60.4%) when the screening-level false positive rate was set to 10% and all positive screens in HCC cases were considered to be true positive screens. By comparison, within this VA cohort, which is a more realistic setting for HCC screening, we observed a 9.76% improvement in the PEB algorithm compared to the standard thresholding approach (63.64% vs 53.88%). The within- and between-subject variability of AFP across control patients in these study populations could explain the difference in performance. In the HALT-C trial, the between-subject variance of log2(AFP) was 1.77, the within-subject variance was 0.39 and the resulting intra-class correlation (ratio of between-subject variance to total variance) was 0.82. In the VA cohort, the between-subject variance was 1.90, the within-subject variance was 0.71 and the intra-class correlation was then 0.73. Therefore, in the VA cohort we observed almost twice the variability in the longitudinal AFP measurements within a patient compared to the HALT-C Trial in patients that do not develop HCC. In this study we do not have information regarding the brand of AFP assay kits used, but this could be a source of the additional variability observed in the VA cohort that we are unable to quantify.

We explored multiple extensions of the PEB algorithm in the VA cohort, including using demographic variables and other liver function markers to explain the variability of AFP, however none of these approaches resulted in clinically significant improvements in the screening performance over the standard PEB algorithm with AFP only (see Additional file 1: Appendix B). The sensitivity of the PEB algorithm also depends on the biomarker behavior after HCC onset, as well as the likelihood of having a screening test soon after HCC onset. In the HALT-C Trial, patients had AFP tests every three months during the first 48 months post-randomization and every six months thereafter. An exploratory analysis that considered only those AFP tests from the HALT-C Trial that were six months apart found that the PEB algorithm method improved the sensitivity of AFP by 12.6% compared to the standard thresholding approach when the screening-level false positive rate was set to 10% and all positive screens in HCC cases were considered to be true positive screens. In the VA cohort, the average time between AFP tests was around 12 months. If we restrict our analysis to only those VA patients with frequent AFP tests (no more than nine months between AFP tests) then the improvement of the PEB algorithm with AFP compared to the standard thresholding approach was 4.37% (57.28% vs 52.91%) when the screening-level false positive rate was set to 10% and all positive screens in HCC cases were considered to be true positive screens.

There are several limitation of this study. The current VA cohort is restricted to those patients with active HCV related cirrhosis and therefore we do not know how these screening algorithms will perform in patients with other etiologies. In addition, the VA patient population in general is older, overwhelming male with few Hispanics and Asians and with high rates of comorbid conditions including alcohol abuse; therefore we do not know how well results generalize to the cirrhosis population in the United States. We are assembling an updated cohort of cirrhosis patients from the VA (2010–2015) that will include multiple cirrhosis etiologies, including HCV, hepatitis B infection, alcoholic liver disease and non-alcoholic fatty liver disease. Patients with non-HCV etiologies have been shown to have lower risk of progression to HCC [17] and in this patient population, we can study the performance of the screening approaches in different cirrhosis subgroups and tailor the algorithms, if necessary, to each disease etiology. We will also study the screening approaches in an external cohort of cirrhosis patients from the community-based Kaiser Permanente Northern California health care system. In this cohort, we will have a more representative sample of the general cirrhosis population in which to further study the screening approaches that we have developed.

## Conclusions

We have evaluated multiple screening algorithms from different perspectives to better understand the potential performance in a future prospective study. In addition, we have extended the definitions of the standard measures (sensitivity, specificity, positive and negative predictive value) from those used when a biomarker is measured at a single time point to the longitudinal screening setting. The proposed measures reflect clinically relevant performance characteristics of the screening algorithms that allow for a clearer understanding of potential future performance.

## Additional file

Additional file 1 Supplementary Materials. Appendix A: Estimators of measures used to evaluate screening algorithms defined in the Methods section. Appendix B: Additional results including Table A–C and Figure A–C. (PDF 267 kb)

## Abbreviations

AASLD: American Association for the Study of Liver Diseases
AFP: α-Fetoprotein
ALT: Alanine aminotransferase
CT: Computed tomography
FPR: False positive rate
HALT-C: Hepatitis C antiviral long-term treatment against cirrhosis
HCC: Hepatocellular carcinoma
HCV: Hepatitis C virus infection
MRI: Magnetic resonance imaging
NPV: Negative predictive value
PEB: Parametric empirical Bayes
PLT: Platelets
PPV: Positive predictive value
TPR: True positive rate
VA: Department of Veterans Affairs
