# Supplementary material for: Evaluating screening approaches for hepatocellular carcinoma in a cohort of HCV related cirrhosis patients from the Veteran’s Affairs Health Care System
Source: BMC Med Res Methodol. 2018 Jan 4;18:1. doi: 10.1186/s12874-017-0458-6 (PMC5753461; doi:10.1186/s12874-017-0458-6)
Supplement: Supplementary file 1 — Supplementary Materials. Appendix A: Estimators of measures used to evaluate screening algorithms defined in the Methods section. Appendix B: Additional results including Table A–C and Figure A–C. (PDF 267 kb) [file 12874_2017_458_MOESM1_ESM.pdf]

# Supplementary Materials: Evaluating screening approaches for hepatocellular carcinoma in a cohort of HCV related cirrhosis patients from the Veteran's Affairs Health Care System

# Appendix A Estimators of measures used to evaluate screening algorithms

For completeness, we include the estimators of the patient-level true positive rate (TPR), screening-level false positive rate (FPR) and positive predictive value that we use in our analysis.

$$\widehat{TPR}(\cdot, \tau_1, \tau_2) = \frac{\sum_i \delta_i I\left[\left\{\sum_{j=1}^{n_i} I(d_i - \tau_1 \le t_{ij} \le d_i - \tau_2) P_{ij}(\cdot)\right\} > 0\right]}{\sum_i \delta_i I\left[\left\{\sum_{j=1}^{n_i} I(d_i - \tau_1 \le t_{ij} \le d_i - \tau_2)\right\} > 0\right]}$$

$$\widehat{FPR}(\cdot, \tau_1) = \frac{\sum_i \sum_{j=1}^{n_i} \{\delta_i I(t_{ij} < d_i - \tau_1) + (1 - \delta_i)\} P_{ij}(\cdot)}{\sum_i \sum_{j=1}^{n_i} \{\delta_i I(t_{ij} < d_i - \tau_1) + (1 - \delta_i)\}}$$

$$\widehat{PPV}(\cdot, \tau_1, \tau_2) = \frac{\sum_i \sum_{j=1}^{n_i} \delta_i I(d_i - \tau_1 \le t_{ij} \le d_i - \tau_2) P_{ij}(\cdot)}{\sum_i \sum_{j=1}^{n_i} P_{ij}(\cdot)}$$

$$\widehat{NPV}(\cdot, \tau_1) = \frac{\sum_i \sum_{j=1}^{n_i} \{\delta_i I(t_{ij} < d_i - \tau_1) + (1 - \delta_i)\} \{1 - P_{ij}(\cdot)\}}{\sum_i \sum_{j=1}^{n_i} \{1 - P_{ij}(\cdot)\}}$$

# Appendix B Additional results

Table A: Comparison of the patient-level true positive fraction ($TPR(\cdot, \tau_1, \tau_2)$) when the threshold for each screening algorithm is chosen such that the screening-level false positive rate is 5% $FPR(\cdot, \tau_1) = 0.05$. In each definition, the choice of the parameters $\tau_1$ and $\tau_2$ varies. A1: $\tau_1 = 6$ months and $\tau_2 = 0$, B1: $\tau_1 = 12$ months and $\tau_2 = 0$, C1: $\tau_1 = 24$ months and $\tau_2 = 0$, D1: $\tau_1$ is the maximum follow-up time and $\tau_2 = 0$. A2: $\tau_1 = 6$ months and $\tau_2 = 3$ months, B2: $\tau_1 = 12$ months and $\tau_2 = 3$ months, C2: $\tau_1 = 24$ months and $\tau_2 = 3$ months, D2: $\tau_1$ is the maximum follow-up time and $\tau_2 = 3$ months. AFP+Lab+ΔAFP: updated laboratory-based algorithm, PEB: AFP: parametric empirical Bayes algorithm applied to AFP.

| | Results from validation cohort | | | | | | | |
|---|---|---|---|---|---|---|---|---|
| Screening algorithm | A1 | B1 | C1 | D1 | A2 | B2 | C2 | D2 |
| AFP only | 0.5123 | 0.4925 | 0.4767 | 0.4701 | 0.3271 | 0.3018 | 0.2640 | 0.2486 |
| AFP+Lab+ΔAFP | 0.5260 | 0.5124 | 0.4860 | 0.4789 | 0.3832 | 0.3829 | 0.3168 | 0.2869 |
| PEB: AFP | 0.5562 | 0.5473 | 0.5395 | 0.5432 | 0.3832 | 0.3829 | 0.3366 | 0.3415 |
| Number of HCC cases | 365 | 402 | 430 | 451 | 107 | 222 | 303 | 366 |

Table B: Sensitivity Analyses: An out-of-bag Bootstrap validation comparing the patient-level true positive fraction ($TPR(\cdot, \tau_1, \tau_2)$) when the threshold for each screening algorithm is chosen such that the screening-level false positive rate is 5% $FPR(\cdot, \tau_1) = 0.05$. In each definition, the choice of the parameters $\tau_1$ and $\tau_2$ varies. A1: $\tau_1 = 6$ months and $\tau_2 = 0$, B1: $\tau_1 = 12$ months and $\tau_2 = 0$, C1: $\tau_1 = 24$ months and $\tau_2 = 0$, D1: $\tau_1$ is the maximum follow-up time and $\tau_2 = 0$. A2: $\tau_1 = 6$ months and $\tau_2 = 3$ months, B2: $\tau_1 = 12$ months and $\tau_2 = 3$ months, C2: $\tau_1 = 24$ months and $\tau_2 = 3$ months, D2: $\tau_1$ is the maximum follow-up time and $\tau_2 = 3$ months. AFP+Lab+ΔAFP: updated laboratory-based algorithm, PEB: AFP: parametric empirical Bayes algorithm applied to AFP.

| | Average results over 300 bootstrap validation cohorts | | | | | | | |
|---|---|---|---|---|---|---|---|---|
| Screening algorithm | A1 | B1 | C1 | D1 | A2 | B2 | C2 | D2 |
| AFP only | 0.5836 | 0.5745 | 0.5623 | 0.5645 | 0.4233 | 0.3977 | 0.3586 | 0.3554 |
| AFP+Lab+ΔAFP | 0.6121 | 0.6051 | 0.5969 | 0.5965 | 0.4635 | 0.4487 | 0.4068 | 0.3904 |
| PEB: AFP | 0.6140 | 0.6073 | 0.6126 | 0.6400 | 0.4146 | 0.4101 | 0.3942 | 0.4267 |

## Alternative parametric empirical Bayes (PEB) approaches

We explored multiple extensions of the PEB algorithm in the VA cohort to determine if incorporating additional patient information, improves the screening performance. The first modification of the PEB algorithm uses the linear predictor of a six-month risk prediction model as the biomarker ($Y_{ij}$). The risk prediction model is a simplification of the risk model in the laboratory-based algorithm and includes $\log_2$(AFP), $\log_2$(ALT), PLT, age at AFP test and two-way interactions between $\log_2$(AFP) and $\log_2$(ALT) and $\log_2$(AFP) and PLT. The model is fit in the testing data using generalized estimating equations with a working correlation matrix that assumes independence and a sandwich variance estimator. This approach is referred to as the "PEB with Gastro 2014" screening algorithm in the results that follow.

The second modification to the PEB algorithm incorporates longitudinal $\log_2$(ALT) and PLT into the PEB algorithm through the hierarchical model assumed for $Y_{ij} = \log_2(AFP_{ij})$

in control patients through the mean structure as follows:

$$Y_{ij}|\theta_i \sim N(\theta_i + \beta_1 \log_2(ALT_{ij}) + \beta_2 PLT_{ij}, \sigma^2)$$
$$\theta_i \sim N(\bar{\theta}, \tau^2).$$

The parameters $\bar{\theta}$, $\beta_1$, $\beta_2$, $\sigma^2$ and $\tau^2$ can be estimated by fitting a linear mixed model with random intercept in the testing data. This approach is referred to as the "PEB with Adjusted AFP" in the results that follow.

The third and fourth modifications of the PEB algorithm allow both the mean and the variance components of the hierarchical model to depend on covariates. For $k = 1, \ldots K$, we assume the following hierarchical model within each subgroup:

$$Y_{ij}|\theta_{ik} \sim N(\theta_{ik}, \sigma_k^2)$$
$$\theta_{ik} \sim N(\bar{\theta}_k, \tau_k^2).$$

The parameters can be estimated by fitting a linear mixed model with random intercept within each subgroup in the testing data. In the third modification of the PEB algorithm, the subgroups are defined based on demographic covariates age and race as follows:

| | White | Black | Other/Unknown |
|---|---|---|---|
| $Age_{ij} < 50$ | k=1 | k=2 | k=3 |
| $50 \leq Age_{ij} < 55$ | k=4 | k=5 | k=6 |
| $55 \leq Age_{ij} < 60$ | k=7 | k=8 | k=9 |
| $60 \leq Age_{ij}$ | k=10 | k=11 | k=12 |

The four age categories are based on approximate quartiles. This approach is referred to as the "PEB with Age and Race" in the results that follow. In the fourth modification of the PEB algorithm, the subgroups are defined based on ALT and PLT levels as follows:

| | $PLT_{ij} < 75$ | $75 \leq PLT_{ij} < 110$ | $110 \leq PLT_{ij} < 170$ | $170 \leq PLT_{ij}$ |
|---|---|---|---|---|
| $\log_2(ALT_{ij}) < 5$ | k=1 | k=2 | k=3 | k=4 |
| $5 \leq \log_2(ALT_{ij}) < 6$ | k=5 | k=6 | k=7 | k=8 |
| $6 \leq \log_2(ALT_{ij}) < 7$ | k=9 | k=10 | k=11 | k=12 |
| $7 \leq \log_2(ALT_{ij})$ | k=13 | k=14 | k=15 | k=16 |

The four ALT and PLT categories are based on approximate quartiles. This approach is referred to as the "PEB with ALT and PLT" in the results that follow.

In Table C, the screening-level FPR is fixed at 10% and in Table D, the screening-level FPR is fixed at 5%. There is some indication that the "PEB with Adjusted AFP" does increase the patient-level true positive rate when only screenings within six months (A1/A2) or one year (B1/B2) are considered true positive screens by a small amount (mostly 1-2%) compared to the original PEB algorithm. However for the other definitions, there is no indication of any difference between the modifications to the PEB algorithm and the original PEB algorithm.

Table C: Comparison of the patient-level true positive rate ($TPR(\cdot, \tau_1, \tau_2)$) when the threshold for each screening algorithm is chosen such that the screening-level false positive rate is 10%, i.e $FPR(\cdot, \tau_1) = 0.1$. In each definition, the choice of the parameters $\tau_1$ and $\tau_2$ varies. A1: $\tau_1 = 6$ months and $\tau_2 = 0$, B1: $\tau_1 = 12$ months and $\tau_2 = 0$, C1: $\tau_1 = 24$ months and $\tau_2 = 0$, D1: $\tau_1$ is the maximum follow-up time and $\tau_2 = 0$. A2: $\tau_1 = 6$ months and $\tau_2 = 3$ months, B2: $\tau_1 = 12$ months and $\tau_2 = 3$ months, C2: $\tau_1 = 24$ months and $\tau_2 = 3$ months, D2: $\tau_1$ is the maximum follow-up time and $\tau_2 = 3$ months.

| | Results from validation cohort | | | | | | | |
|---|---|---|---|---|---|---|---|---|
| Screening algorithm | A1 | B1 | C1 | D1 | A2 | B2 | C2 | D2 |
| PEB | 0.6055 | 0.6045 | 0.6000 | 0.6364 | 0.4579 | 0.4955 | 0.4620 | 0.4891 |
| PEB with Gastro 2014 | 0.6247 | 0.6318 | 0.6233 | 0.6341 | 0.5047 | 0.5135 | 0.4554 | 0.4508 |
| PEB with Adjusted AFP | 0.6247 | 0.6219 | 0.6116 | 0.6319 | 0.4953 | 0.5045 | 0.4554 | 0.4672 |
| PEB with Age and Race | 0.6055 | 0.6020 | 0.5977 | 0.6319 | 0.4673 | 0.5000 | 0.4554 | 0.4809 |
| PEB with ALT and PLT | 0.6164 | 0.6119 | 0.6140 | 0.6231 | 0.4766 | 0.4820 | 0.4587 | 0.4508 |
| Number of HCC cases | 365 | 402 | 430 | 451 | 107 | 222 | 303 | 366 |

Table D: Comparison of the patient-level true positive rate ($TPR(\cdot, \tau_1, \tau_2)$) when the threshold for each screening algorithm is chosen such that the screening-level false positive rate is 5%, i.e $FPR(\cdot, \tau_1) = 0.1$. In each definition, the choice of the parameters $\tau_1$ and $\tau_2$ varies. A1: $\tau_1 = 6$ months and $\tau_2 = 0$, B1: $\tau_1 = 12$ months and $\tau_2 = 0$, C1: $\tau_1 = 24$ months and $\tau_2 = 0$, D1: $\tau_1$ is the maximum follow-up time and $\tau_2 = 0$. A2: $\tau_1 = 6$ months and $\tau_2 = 3$ months, B2: $\tau_1 = 12$ months and $\tau_2 = 3$ months, C2: $\tau_1 = 24$ months and $\tau_2 = 3$ months, D2: $\tau_1$ is the maximum follow-up time and $\tau_2 = 3$ months.

| | Results from validation cohort | | | | | | | |
|---|---|---|---|---|---|---|---|---|
| Screening algorithm | A1 | B1 | C1 | D1 | A2 | B2 | C2 | D2 |
| PEB | 0.5562 | 0.5498 | 0.5395 | 0.5455 | 0.3832 | 0.3829 | 0.3366 | 0.3415 |
| PEB with Gastro 2014 | 0.5534 | 0.5473 | 0.5302 | 0.5277 | 0.4112 | 0.4144 | 0.3564 | 0.3306 |
| PEB with Adjusted AFP | 0.5671 | 0.5547 | 0.5395 | 0.5410 | 0.4019 | 0.4009 | 0.3498 | 0.3388 |
| PEB with Age and Race | 0.5507 | 0.5448 | 0.5395 | 0.5432 | 0.3925 | 0.3919 | 0.3498 | 0.3415 |
| PEB with ALT and PLT | 0.5589 | 0.5473 | 0.5372 | 0.5410 | 0.4112 | 0.3919 | 0.3432 | 0.3279 |
| Number of HCC cases | 365 | 402 | 430 | 451 | 107 | 222 | 303 | 366 |

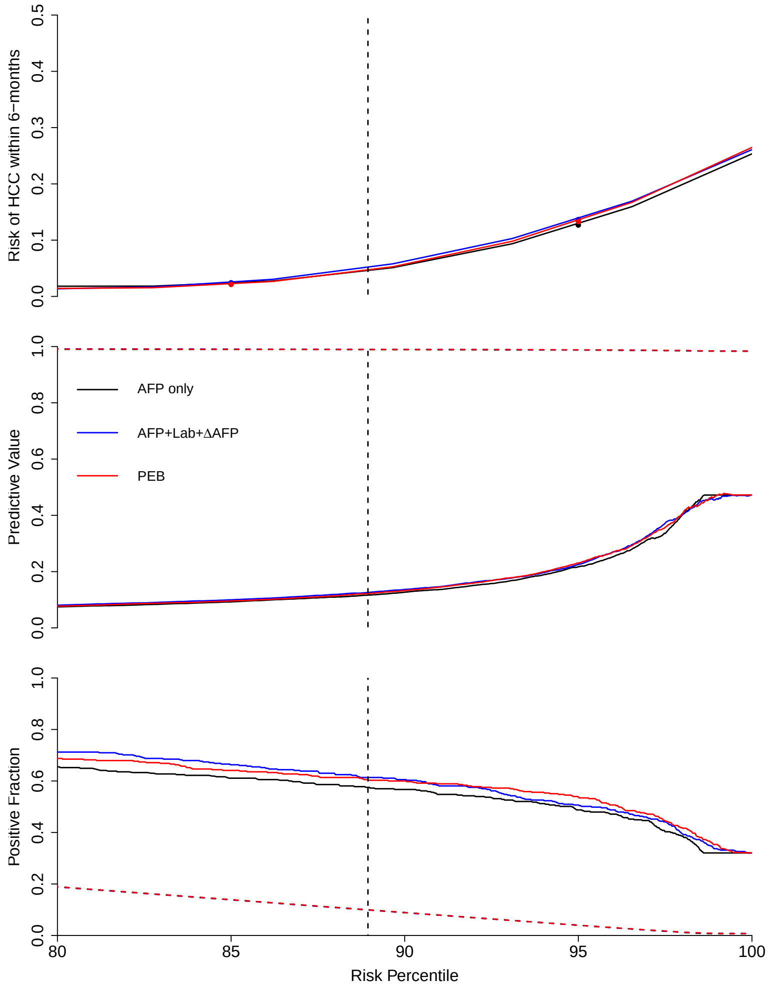


Figure A: Comparison of screening algorithms within six months of clinical diagnosis (A1 in Figure 3). In the top panel, we plot the estimated risk of HCC within two years for each screening approach against corresponding the risk percentile, which is defined to be the corresponding proportion of screens that lie below the threshold. The middle panel displays the positive predictive value ($PPV(\cdot, \tau_1 = 24, \tau_2 = 0)$, solid line) and the negative predictive value ($NPV(\cdot, \tau_1 = 24)$, dashed line) against the risk percentile and the bottom panel displays the patient-level true positive fraction ($TPR(\cdot, \tau_1 = 24, \tau_2 = 0)$, solid line) and the screening-level false positive fraction ($FPR(\cdot, \tau_1 = 24)$, dashed line) against the risk percentile. The vertical dashed lines in each plot correspond to the risk percentile associated with 10% screening-level FPR. The figures focus on curves between the 80th and 100th risk percentile. AFP+Lab+ΔAFP: updated laboratory-based algorithm, PEB: AFP: parametric empirical Bayes algorithm applied to AFP.

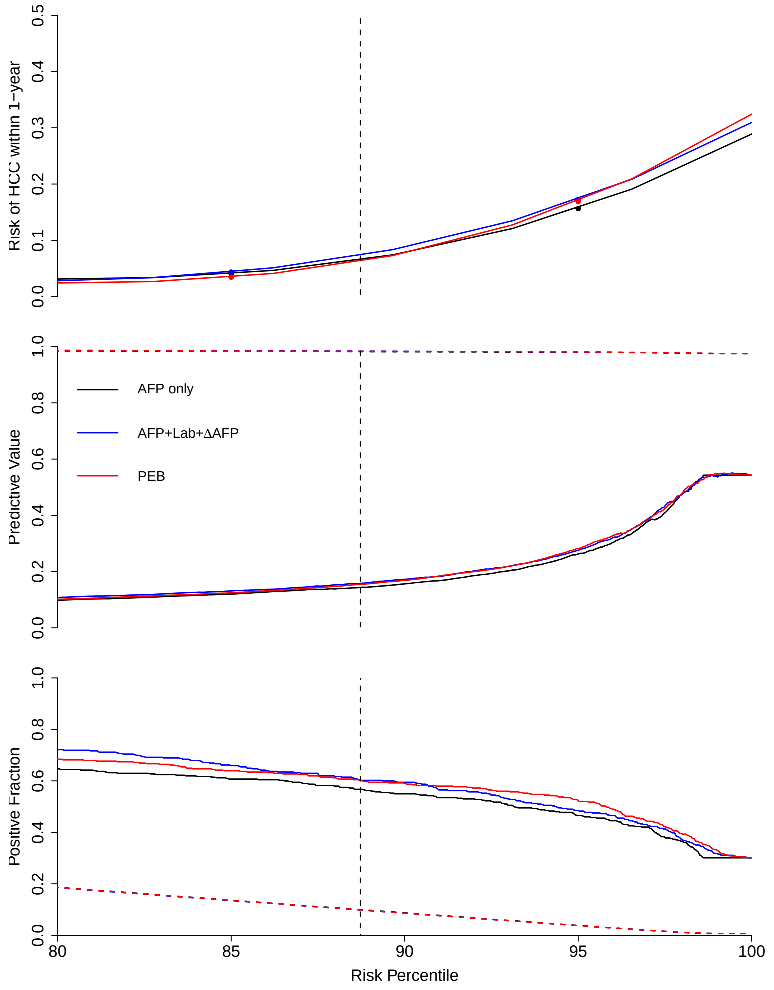


Figure B: Comparison of screening algorithms within one year of clinical diagnosis (B1 in Figure 3). In the top panel, we plot the estimated risk of HCC within two years for each screening approach against corresponding the risk percentile, which is defined to be the corresponding proportion of screens that lie below the threshold. The middle panel displays the positive predictive value ($PPV(\cdot, \tau_1 = 24, \tau_2 = 0)$, solid line) and the negative predictive value ($NPV(\cdot, \tau_1 = 24)$, dashed line) against the risk percentile and the bottom panel displays the patient-level true positive fraction ($TPR(\cdot, \tau_1 = 24, \tau_2 = 0)$, solid line) and the screening-level false positive fraction ($FPR(\cdot, \tau_1 = 24)$, dashed line) against the risk percentile. The vertical dashed lines in each plot correspond to the risk percentile associated with 10% screening-level FPR. The figures focus on curves between the 80th and 100th risk percentile. AFP+Lab+ΔAFP: updated laboratory-based algorithm, PEB: AFP: parametric empirical Bayes algorithm applied to AFP.

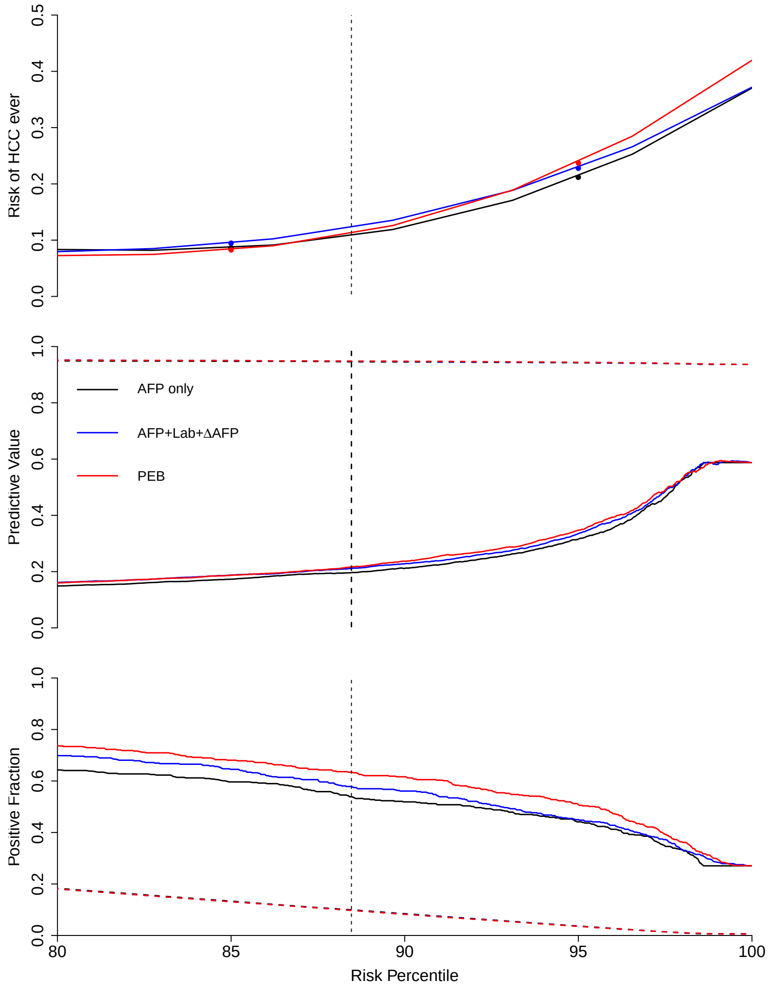


Figure C: Comparison of screening algorithms across the entire screening period (D1 in Figure 3). In the top panel, we plot the estimated risk of HCC within two years for each screening approach against corresponding the risk percentile, which is defined to be the corresponding proportion of screens that lie below the threshold. The middle panel displays the positive predictive value ($PPV(\cdot, \tau_1 = 24, \tau_2 = 0)$, solid line) and the negative predictive value ($NPV(\cdot, \tau_1 = 24)$, dashed line) against the risk percentile and the bottom panel displays the patient-level true positive fraction ($TPR(\cdot, \tau_1 = 24, \tau_2 = 0)$, solid line) and the screening-level false positive fraction ($FPR(\cdot, \tau_1 = 24)$, dashed line) against the risk percentile. The vertical dashed lines in each plot correspond to the risk percentile associated with 10% screening-level FPR. The figures focus on curves between the 80th and 100th risk percentile. AFP+Lab+ΔAFP: updated laboratory-based algorithm, PEB: AFP: parametric empirical Bayes algorithm applied to AFP.
